# Comparison between a serum creatinine-and a cystatin C-based glomerular filtration rate equation in patients receiving amphotericin B

**DOI:** 10.1186/s40199-016-0149-6

**Published:** 2016-06-06

**Authors:** Iman Karimzadeh, Hossein Khalili

**Affiliations:** Department of Clinical Pharmacy, Faculty of Pharmacy, Shiraz University of Medical Sciences, Shiraz, Iran; Department of Clinical Pharmacy, Faculty of Pharmacy, Tehran University of Medical Sciences, Enghelab Ave, Tehran, Iran

**Keywords:** Serum cystatin C, Serum creatinine, Glomerular filtration rate, Amphotericin B

## Abstract

Serum cystatin C (Cys C) has a number of advantages over serum creatinine in the evaluation of kidney function. Apart from Cys C level itself, several formulas have also been introduced in different clinical settings for the estimation of glomerular filtration rate (GFR) based upon serum Cys C level. The aim of the present study was to compare a serum Cys C-based equation with Cockcroft-Gault serum creatinine-based formula, both used in the calculation of GFR, in patients receiving amphotericin B. Fifty four adult patients with no history of acute or chronic kidney injury having been planned to receive conventional amphotericin B for an anticipated duration of at least 1 week for any indication were recruited. At three time points during amphotericin B treatment, including days 0, 7, and 14, serum cystatin C as well as creatinine levels were measured. GFR at the above time points was estimated by both creatinine (Cockcroft-Gault) and serum Cys C based equations. There was significant correlation between creatinine-based and Cys C-based GFR values at days 0 (*R* = 0.606, *P* = 0.001) and 7 (*R* = 0.714, *P* < 0.001). In contrast to GFR estimated by the Cockcroft-Gault equation, the mean (95 % confidence interval) Cys C-based GFR values at different studied time points were comparable within as well as between patients with and without amphotericin B nephrotoxicity. Our results suggested that the Gentian Cys C-based GFR equation correlated significantly with the Cockcroft-Gault formula at least at the early time period of treatment with amphotericin B.

**Graphical abstract:**
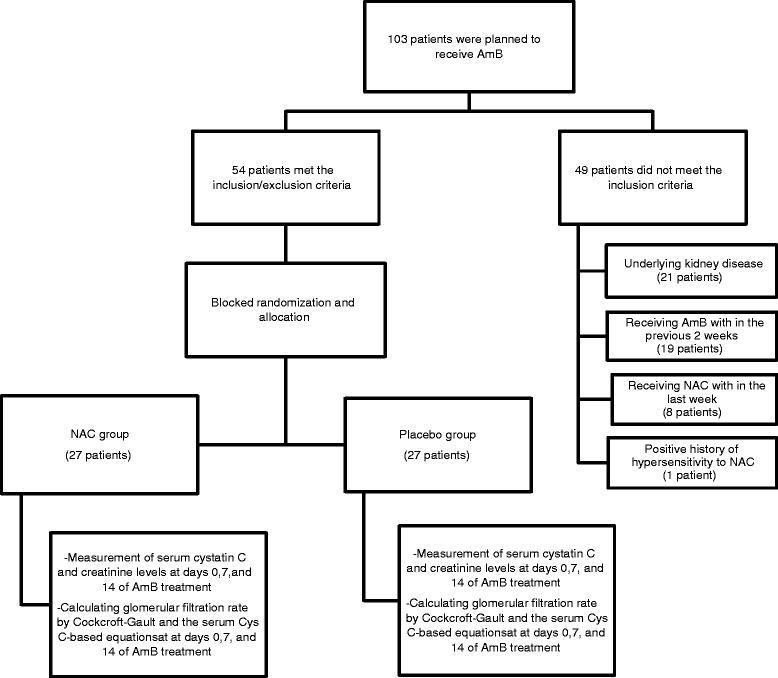
Comparison between a serum creatinine-and a cystatin C-based glomerular filtration rate equation in patients receiving amphotericin B

Comparison between a serum creatinine-and a cystatin C-based glomerular filtration rate equation in patients receiving amphotericin B

## Introduction

Serum cystatin C (Cys C), a 13 kDa non-glycosylated protein with cysteine protease inhibitor activity, has been proposed as an alternative marker to creatinine for assessing kidney function [1]. It lacks a number of serum creatinine drawbacks such as being influenced by non-renal factors including age, gender, muscle mass, and physical activity [2, 3]. Dose adjustment of many medications such as antibacterials depends on patients’ glomerular filtration rate (GFR). Direct measurement of GFR, using urinary inulin clearance and the plasma ^99m^Tc-DTPA or ^125^-iothalamate is cumbersome, costly, and not readily available [4].

Besides Cys C level itself, different formulas have also been introduced in different clinical settings such as kidney transplant recipients [5], critically ill patients [6], chronic kidney disease [7], newborns [8], and the elderly [9] for the estimation of GFR, based upon Cys C serum level. In contrast to Cockcroft-Gault (CG) and Modification of Diet in Renal Disease (MDRD) formulas, which need several variables such as age and sex for calculation, Cys C-based equations are mainly dependent only on serum Cys C levels [10]. To best of our knowledge, these equations have not been investigated well enough in drug-induced acute kidney injury (AKI) conditions. The aim of the present preliminary study was to compare a serum Cys C-based equation with the classic and prominent CG serum creatinine-based formula, both used for the calculation of GFR, in patients receiving amphotericin B (AmB).

## Methods

The data of this study was extracted from a multicentre randomized, double-blinded, placebo-controlled, clinical trial (ID: IRCT201107233449N8) that assessed the effectiveness of oral N-acetylcysteine (NAC) co-treatment with AmB in preventing major features of AmB nephrotoxicity [11]. Carried out in a 15-months period, from early August 2012 to November 2013, at three university health-care settings affiliated to Tehran University of Medical Sciences, Tehran, Iran, the study included 54 adult individuals with no documented history of AKI or chronic kidney disease, having been planned to receive conventional AmB for an anticipated duration of at least 1 week for any indication. They were given either placebo or 600 mg oral NAC twice daily during the treatment course of AmB. The institutional review boards and the medical ethics committees of all hospitals approved the study and all patients or their family members signed and approved a written informed consent form.

At days 0, 7, and 14 of AmB treatment, serum Cys C as well as creatinine levels were measured. Serum creatinine level was determined by an Auto-analyzer (Biotechnica BT-3000, Italy) based on the modified Jaffe colorimetric reaction. Serum Cys C level was measured by the turbidimetric method (Gentian, Moss, Norway). GFR at days 0, 7, and 14 was calculated by the CG formula [(140–age) × (Body weight) × (0.85 if female)/(serum creatinine × 72)] [12]. CG values were adjusted by body surface area of relevant patients and reported as ml/min/1.73 m^2^. Besides CG, GFR at the above time points was also estimated by the serum Cys C-based equation, provided in the package insert of Gentian assay kit (79.901/Serum Cys C^1.4389^) [13]. AmB nephrotoxicity was defined by either a 50 % or more decline in the estimated GFR according to the CG formula or the doubling of serum creatinine from the baseline values [14].

### Statistical analyses

The possible correlation between creatinine-based and Cys C-based GFR values at days 0, 7, and 14 were assessed by the Pearson correlation test. Comparison of the mean values (95 % confidence interval [CI]) of calculated creatinine-based as well as Cys C-based GFR at the above time points within and between patients with and without AmB nephrotoxicity was done by the one-way analysis of variance (ANOVA) with repeated measures. *P* values < 0.05 were considered statistically significant. Statistical analyses were carried out by the SPSS (Statistical Package for the Social Sciences) version 20 software.

## Results

Among 54 patients randomly allocated into either placebo or NAC receiving group, 23 (42.59 %) developed AmB nephrotoxicity. The mean ± standard deviation creatinine-based GFR values at days 0, 7, and 14 were 92.94 ± 42.04, 92.21 ± 45.92, and 54.29 ± 20.63 ml/min/1.73 m^2^, respectively. The Cys C-based GFR value was 73.66 ± 34.24 ml/min/1.73 m^2^ at day 0, 78.19 ± 41.37 ml/min/1.73 m^2^ at day 7, and 58.36 ± 25.76 ml/min/1.73 m^2^ at day 14.

**Fig. 1:**
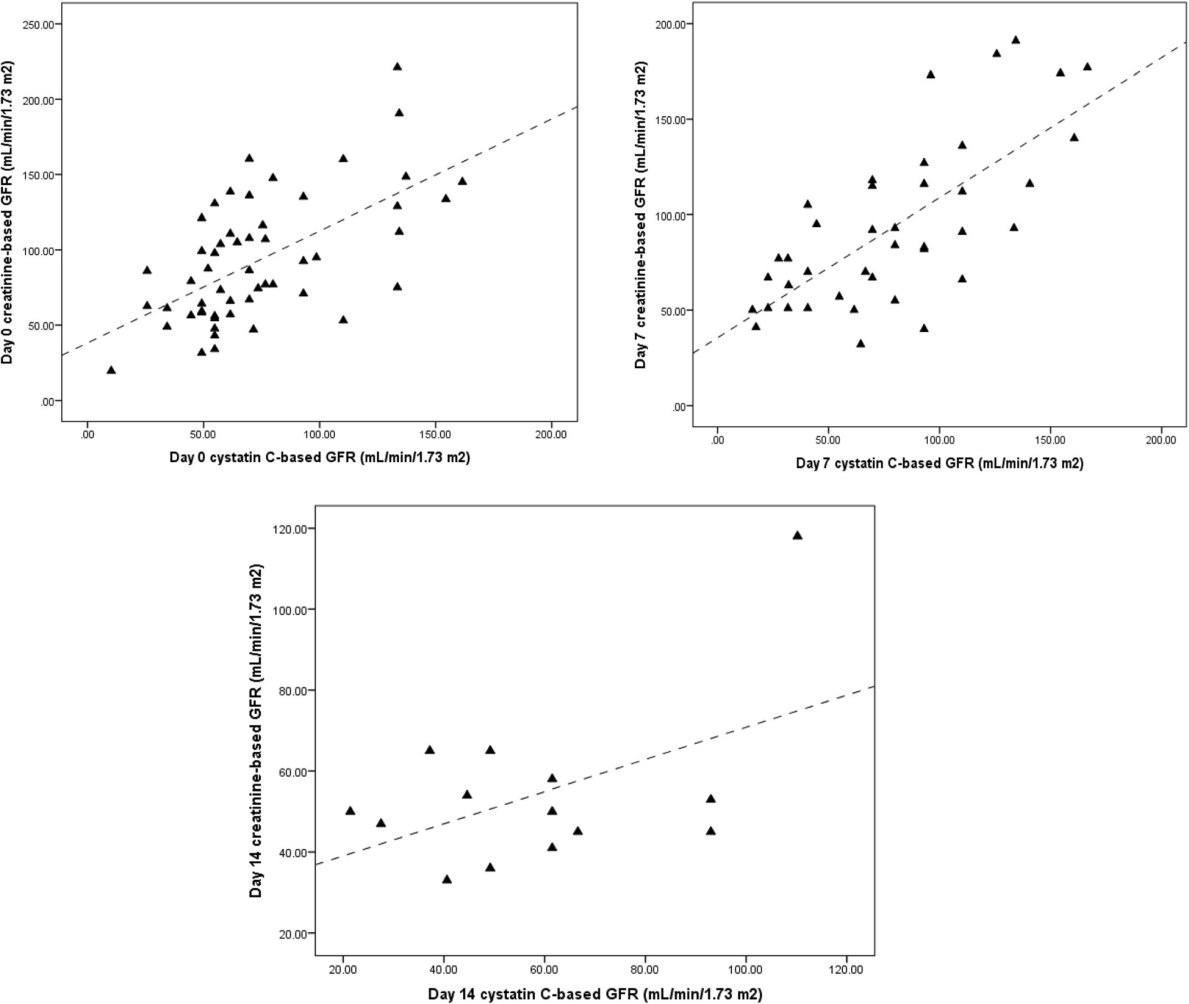
The possible correlation between serum creatinine-and serum cystatin C-based GFR values at days 0, 7, 14 of amphotericin B treatment

As depicted in Fig. 1, there was significant correlation between creatinine-based and Cys C-based GFR values at days 0 (*R* = 0.606, *P* = 0.001) and 7 (*R* = 0.714, *P* < 0.001). In contrast, the correlation of these values at day 14 was not statistically significant (*R* = 0.496, *P* < 0.071).

**Fig. 2:**
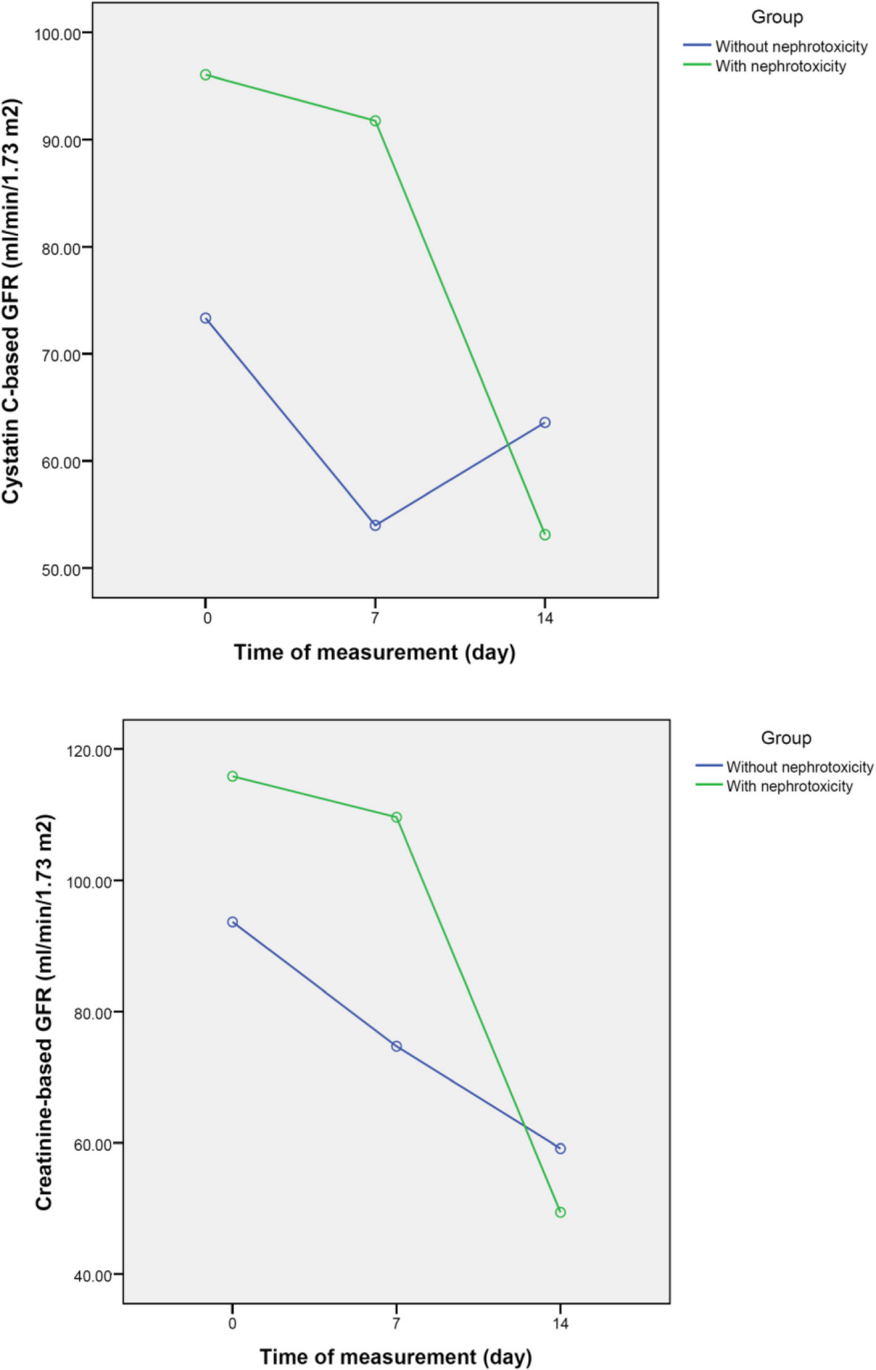
Changing pattern in creatinine-and serum cystatin C-based GFR values at three time points in patients with and without amphotericin B nephrotoxicity

According to results of ANOVA with repeated measure analysis (Table 1 & Fig. 2), the mean (95 % CI) creatinine-based GFR at day 14 was significantly lower than that at day 7 in patients who developed AmB nephrotoxicity (*P* = 0.024). Furthermore, the mean (95 % CI) decrease in creatinine-based GFR values at day 14 compared to day 0 (−50.457 [−89.477 to-11.437] ml/min/1.73 m^2^) as well as day 14 versus day 7 (−37.857 [−63.514 to−12.2] ml/min/1.73 m^2^), were statistically significant between individuals with and without AmB nephrotoxicity (*P* = 0.016 and *P* = 0.007, respectively). In contrast to creatinine-based calculated GFR, the mean (95 % CI) Cys C-based GFR values at different studied time points were comparable within as well as between patients with and without AmB nephrotoxicity.

**Table 1:** Mean (95 % confidence interval) changes of creatinine-and serum cystatin C-based GFR values at days 0, 7 and 14 of amphotericin B treatment within and between patients with and without AmB nephrotoxicity

Time point	Day 7 vs. Day 0	Day 14 vs. Day 0	Day 14 vs. Day 7
Creatinine-based GFR (ml/min/1.73 m^2^)			
Mean (95 % confidence interval) difference values in patients with nephrotoxicity [*P* value]	-6.258 (−82.997 to 70.480) [1]	-66.401 (−150.289 to 17.487) [0.122]	-60.143 (−110.816 to-9.470) [0.024]
Mean (95 % confidence interval) difference values in patients without nephrotoxicity [*P* value]	-18.942 (−70.939 to 33.055) [0.829]	-34.514 (−177.143 to 48.116) [0.656]	-15.571 (−74.111 to 42.968) [1]
Mean (95 % confidence interval) difference values between two groups [*P* value]	-12.6 (−43.318 to 18.118) [0.389]	-50.457 (−89.477 to-11.437) [0.016]	-37.857 (−63.514 to−12.2) [0.007]
Cystatin C-based GFR (ml/min/1.73 m^2^)			
Mean (95 % confidence interval) difference values in patients with nephrotoxicity [*P* value]	-4.3 (−108.050 to 99.45) [1]	-42.943 (− 103.840 to 17.954) [0.179]	-38.643 (−96.965 to 19.679) [0.217]
Mean (95 % confidence interval) difference values in patients without nephrotoxicity [*P* value]	-19.357 (−59.401 to 20.686) [0.489]	-9.729 (−56.979 to 37.521) [1]	9.629 (−48.990 to 68.247) [1]
Mean (95 % confidence interval) difference values between two groups [*P* value]	-11.829 (−48.682 to 25.024) [0.498]	-26.336 (−51.878 to - 0.793) [0.44]	-14.507 (–41.909 to 12.895) [0.271]

## Discussion

Although studied extensively, Cys C-based GFR equations have not generally been introduced into routine clinical practice yet. Considerable heterogeneity between relevant GFR equations can be partially taken into account for this matter [15]. Substantial heterogeneity between Cys C-based GFR equations can be in turn attributed to four major factors including: (1) study population differences, (2) different gold standard methods of GFR measurements, (3) lack of international standardized calibration for measurement of Cys C, and (4) variation in exploited analytical techniques as well as reagents [10]. Regarding the first factor involved, elevated body mass index (BMI) can be associated with an increase in the Cys C concentration by about 10 %. Furthermore, serum Cys C concentrations have been reported to be lower in females than males (about 9 %) [15]. In the present study, no gold standard method was used for determining GFR because of both financial and technical problems.

Regarding the last two factors, three major techniques including particle-enhanced nephelometric assay (PENIA), particle enhanced turbidimetric assay (PETIA), and enzyme-linked immunosorbent assay (ELISA) are commonly used for determining Cys C. A meta-analysis on 46 articles published until December 31, 2001, revealed that immunonephelometric methods of Cys C assay produced significantly greater correlations with GFR than other assay methods (*r* = 0.846 versus *r* = 0.784, respectively; *P* < 0.001) [16]. In a study on 80 healthy volunteers and 20 patients with renal and/or heart disease, the mean difference between ELISA and PETIA or ELISA and PENIA was 0.65 ± 0.63 μg/ml and 0.58 ± 0.53 μg/ml, respectively [17]. Interestingly, Tidman et al. demonstrated that serum Cys C concentrations obtained by the Gentian method were approximately 10 % lower than the DAKO method within the normal GFR range. They also reported that among Cys C-based GFR formulas examined in 644 patients, the former Orebro-cyst Gentian equation (100/serum Cys C—14) had the highest accuracy [10].

The Cys C-based GFR equation used in our study was derived from Flodin et al. investigation on 160 patient samples aged above 15 years. Linear regression analysis showed that there was strong correlation between Gentian Cys C assay using a chemistry instrument (Architect ci8200) and iohexol clearance (*R*^2^ = 0.956) [18]. Lack of significant correlation between creatinine-based and Cys C-based GFR values only at day 14 but not days 0 and 7 of AmB treatment in our cohort, may be due to the limited number of patients (only 16) that remained in the study at this time point. It is noteworthy that considering only correlation coefficient in our survey seems inadequate and precision, accuracy, and relative difference should also be calculated to compare these two formulas properly. The pattern of Cys C-based GFR values decreased continuously during the study in patients with AmB nephrotoxicity; but these changes were not statistically significant in contrast to creatinine-based GFR values. This may be justified by the limited number of serum Cys C level measurements at only three time points during AmB treatment, high intraindividual variability of serum Cys C, and absence of a gold standard method for measuring GFR.

In conclusion, our preliminary findings suggested that the Gentian Cys C-based GFR calculation equation correlated significantly with CG formula at least at the early time period of AmB treatment. However, the continuous decreasing trend in the mean (95 % CI) values of Cys C-based GFR at the studied time points was not statistically significant in patients who developed AmB nephrotoxicity. Measuring serum Cys C level at more frequent and closer time points and exploiting a gold standard method for measuring GFR can be considered for future studies in the comparison of serum Cys C-based equations with serum creatinine-based formulas used for the calculation of GFR in patients receiving nephrotoxic medications such as AmB.
